# Association Between Alexithymia and Functional Gastrointestinal Disorders

**DOI:** 10.3389/fpsyg.2018.00599

**Published:** 2018-04-25

**Authors:** Michiko Kano, Yuka Endo, Shin Fukudo

**Affiliations:** ^1^Frontier Research Institute for Interdisciplinary Sciences, Tohoku University, Sendai, Japan; ^2^Behavioral Medicine, Graduated School of Medicine, Tohoku University, Sendai, Japan; ^3^Psychosomatic Medicine, Tohoku University Hospital, Tohoku University, Sendai, Japan

**Keywords:** alexithymia, functional gastrointestinal disorders, Irritable Bowel Syndrome, functional dyspepsia, somatoform amplification

The personality construct alexithymia is characterized by the difficulty in identifying and describing feelings with an externally oriented thinking pattern and a limited imaginative capacity (Nemiah et al., 1976). Alexithymia was first described as a specific cognitive and affective style of patients with classic psychosomatic diseases who showed little insight into their emotions and failed to respond to dynamic psychotherapy (Sifneos, 1967). Studies have found that alexithymia contributes to various medical conditions, including gastrointestinal diseases, cardiovascular diseases, obesity, chronic pain, renal failure, eating disorders, panic disorders, and posttraumatic stress disorders (Taylor et al., 1997). Alexithymia has been recognized as a risk factor for various physical and mental health problems; however, the mechanism that links alexithymia with these physical symptoms remains unclear.

Functional gastrointestinal disorders (FGIDs) are one of the conditions associated with alexithymia that has a high prevalence (Porcelli and Todarello, 2007). FGIDs are characterized by chronically recurring gastrointestinal symptoms in the absence of structural or biochemical abnormalities (Drossman, 2016). FGIDs are defined as disorders of the gut-brain interaction, which is a complex interaction that may be dysregulated by microbial dysbiosis within the gut, altered mucosal immune function, altered gut signaling (visceral hypersensitivity), and central nervous system modulation of gut signaling and motor function (Drossman and Hasler, 2016). FGIDs have been studied from a biopsychosocial perspective and shown that psychological and social factors have an impact on FGIDs (Van Oudenhove et al., 2016). Irritable bowel syndrome (IBS) and functional dyspepsia (FD) are the most widely recognized FGIDs with a prevalence of 11.2% (Lovell and Ford, 2012) and 10–30% (Mahadeva and Goh, 2006) worldwide, respectively.

To identify the association between alexithymia and FGIDs, we searched the relevant papers on PubMed from 1985 until September 2017 for full-text articles with a combination of “alexithymia” and “functional gastrointestinal disorders,” “irritable bowel syndrome,” “functional dyspepsia,” or “gastrointestinal” in the title of abstract. The first version of the Toronto Alexithymia Scale was published in 1985 (Taylor et al., 1985).

## Alexithymia in FGIDs

The studies of alexithymia in FGIDs are summarized in Table 1. A high prevalence of alexithymia has been reported in patients with FGIDs (Porcelli et al., 1999, 2003, 2004b; Mazaheri et al., 2012). Alexithymia was a negative predictor of treatment outcome (failure to improve) in FGIDs (Porcelli et al., 2003, 2004b), while health anxiety (hypochondria) predicted improvement (Porcelli et al., 2004b). Relative to depression, alexithymia was the stronger predictor for poor outcome (Porcelli et al., 2003). In a comparison between FGID patients with comorbid psychopathology and psychiatric outpatients with comorbid FGIDs, gastrointestinal symptoms were not significantly different between groups, but the FGIDs patients with psychopathology were more alexithymic and visited a gastroenterologist (Porcelli et al., 2004a). Alexithymia may contribute to the onset or maintenance of FGIDs independent of psychiatric disorders such as anxiety or depression, and illness behavior to seek medical help.

**Table 1:** Alexithymia and FGIDs.

**Year first author**	**Subjects**	**Alexithymia measure**	**Diagnosis of FGIDs**	**Results**
**FGIDs**				
2012 Mazaheri	129 FGIDs (47 m) 108 Controls (39 m)	TAS-20	ROME III GSRS	Mean scores of alexithymia and its subscales were higher in FGIDs than Controls
2004 Porcelli	58 FGIDs (26FD, 12IBS, 9FD&IBS, 10FAP) 52 psychiatric patients	TAS-20	ROME I	FGIDs had higher alexithymia and more severe gastrointestinal symptoms than psychiatric patients.
2004 Porcelli	118 FGIDs	DCPR	ROME I	In unimproved patients, the prevalence of alexithymia and persistent somatization was higher while healthy anxiety was more prevalent in improved patients.
2003 Porcelli	112 FGIDs (25FD, 25IBS, 8FD&IBS, 10FAP)	TAS-20	ROME I	Base-line alexithymia and depression were significant predictors of treatment outcome in FGID patients.
1999 Porcelli	112 FGIDs (37FD, 29IBS, 20FAP, 35FD&IBS) 116 IBD 112 HC	TAS-20	ROME	The FGID group was significantly more alexithymic than the IBD group, and the two gastrointestinal groups were more alexithymic than the normal healthy group
**IBS**				
2017 Porcelli	150 IBS	TAS-20	ROMEIII	Alexithymia and gastrointestinal-specific anxiety (GSA) were closely related IBS symptoms. Only alexithymia was found to be a stable trait and a stronger predictor of treatment outcome than GSA
2016 Huang	10 adolescents IBS 10 adolescents IBD 10 HC	TAS-20	ROMEIII	TAS-20 score was higher in IBS and IBD than HC. Higher activation within interoceptive brain regions during anticipated pain was observed in IBS compared to IBD and HC subjects. IBD patients demonstrated increased activation in perceptual brain regions during experienced pain as compared to IBS and HC.
2014 Farnam	100 IBS	TAS-20	ROMEIII	IBS patients scored higher in TAS-20 and all three subscales of alexithymia. The level of alexithymia did not influence on the outcome by emotional awareness training.
2014 Porcelli	177 IBS	TAS-20	ROME III	The highest IBS severity scores were obtained by patients with high alexithymia alone or combined with higher GSA.
2013 Phillips	82 IBS 67 controls	TAS-20		Alexithymia and the defectiveness schema related to both IBS and symptom severity.
2011 Endo	256 boys and 335 girls (14 y.o. in 2009)	TAS-20	ROME II Modular Questionnaire	In IBS students (19% of total students), TAS-20 total, DIF, and DDF scores were higher than controls. Alexithymic IBS (TAS-20 > 50) showed higher IBS scores than low alexithymic IBS (TAS20 ≤ 50).
2006 Jones	74 IBS 48 IBD 55 HC	TAS-20	ROME II	Patients with IBS and IBD had significantly higher scores for both alexithymia and somatosensory amplification compared with controls.
2003 Portincasa	100 IBS (27M) 100 HCs (30M)	TAS-20	ROME II	IBS patients had increased scores TAS-20 compared to healthy subjects. Higher alexithymia was found in 43% of IBS patients and in 2% of HCs.
1998 Arun	30 IBS 30 HCs	TAS		More IBS patients were higher alexithymia (127 ≤ TAS).
**FD**				
2005 Jones	103 FDs 67 HCs	TAS-20	ROME II	TAS-20 and DIF scores were higher in FDs.
2004 Jones	111 FDs 53 HCs	TAS-20	ROME II	Higher levels of alexithymia and somatosensory amplification in patients with functional dyspepsia
FCP				
2011 White	231 NCCP (56% females)	TAS-20		Alexithymia and anxiety sensitivity were both uniquely and independently associated with pain severity and life interference due to pain. Alexithymia may be increasingly stable over time.
1997 Lumley	15 NCCP Ischemia 34 Silent ischemia 68	TAS-20		The patients with NCCP and the patients with silent ischemia had higher TAS-20 total than no ischemia/no chest pain patients. The patients with NCCP had higher score of DIF and DDF than the no ischemia/no chest pain patients.

*DCPR, Diagnostic Criteria for Psychosomatic Research; DDF, difficulty describing feelings; DIF, difficulty identifying feelings; F, females; FAP, functional abdominal pain; FCP, functional chest pain; FD, functional dyspepsia; FGIDs, functional gastrointestinal disorders; GSA, gastrointestinal-specific anxiety; GSRS, Gastrointestinal Symptom Rating Scale; HC, healthy controls; IBS, Irritable bowel syndrome; IBD, inflammatory bowel disease; M, males; NCCP, non-cardiac chest pain; TAS, Toronto alexithymia scale*.

In patients with IBS, the prevalence of alexithymia or alexithymia level was high (Arun, 1998; Jones et al., 2006; Endo et al., 2011; Phillips et al., 2013; Farnam et al., 2014; Huang et al., 2016) and IBS severity was positively associated with alexithymia (Endo et al., 2011; Phillips et al., 2013; Porcelli et al., 2014). Furthermore, alexithymia and gastrointestinal-specific anxiety (GAS) were closely related to IBS symptoms (Porcelli et al., 2014, 2017), and the highest IBS severity was associated with alexithymia alone (Porcelli et al., 2014); only alexithymia was found to be a stable trait and a stronger predictor of treatment outcome of IBS (Porcelli et al., 2017). In addition to alexithymia, the same study found that somatosensory amplification, which refers to the tendency to experience somatic sensation as intense, was also higher in patients with IBS (Jones et al., 2006). In one randomized clinical trial to evaluate the therapeutic effect of emotional awareness training, alexithymia did not correlate with the overall outcome of pain severity or pain frequency (Farnam et al., 2014). Thus, alexithymia may be a more reliable trait than GAS and is associated with the severity of IBS.

In two functional dyspepsia studies from the same group, a high level of alexithymia was found in patients with FD (Jones et al., 2004, 2005). Level of somatoform amplification was also higher in patients with FD than in controls, but there was no correlation between somatosensory amplification and alexithymia (Jones et al., 2004).

The alexithymia score was high (Lumley et al., 1996) in patients with non-cardiac chest pain (NCCP), which is now categorized as functional chest pain as part of esophageal disorders of FGIDs (Drossman, 2016), and alexithymia and anxiety sensitivity were both uniquely associated with pain severity (White et al., 2011).

## Alexithymia in other gastrointestinal conditions

Inflammatory bowel disorders (IBD) are classic psychosomatic diseases (Sifneos, 1967; Taylor et al., 1981), and several studies have demonstrated that patients with IBD have high alexithymia (Porcelli et al., 1999; Jones et al., 2006; Huang et al., 2016). In these cases, alexithymia was associated with a poor quality of life (Mazaheri et al., 2012). One study reported that the FGID group was significantly more alexithymic than the IBD group (Porcelli et al., 1999), while another study found that patients with IBS and IBD did not differ from one another in terms of alexithymia severity (Jones et al., 2006). Alexithymia levels were related to the abdominal symptoms, but not with upper endoscopy findings (van Kerkhoven et al., 2006). On the other hand, a previous study demonstrated that alexithymia was higher in the peptic ulcer group than in the erosive gastritis group (Fukunishi et al., 1997), and both adenoma and adenocarcinoma patients had higher alexithymia scores than controls (Lauriola et al., 2011). Interestingly, in a 3-year prospective study with 60 colorectal cancer patients who underwent elective cholecystectomy, the high alexithymia group showed a significantly higher health related quality of life than did the lower alexithymia group during the postoperative period (Ripetti et al., 2008). Alexithymia predicted better outcomes of postoperative psychosocial adjustment several years after pelvic pouch surgery for ulcerative colitis (Weinryb et al., 2003). These studies indicate that alexithymia might be advantageous for psychosocial adaptation after surgery.

## Alexithymia measurement

Most of studies which listed in Table 1 used 20-itemToronto alexithymia scale (TAS-20) (Bagby et al., 1994a,b). The TAS-20 is a self-reported measurement and has been used as a reliable, validated, and common metric for measuring alexithymia in a broad variety of studies (Lumley et al., 2007). On the other hand, there is an argument that TAS-20 tends to correlate with negative affect, such as anxiety and depression, and it is sometimes difficult to distinguish the influence of negative emotions from that of alexithymia on the clinical conditions (Subic-Wrana et al., 2005). The Levels of Emotional Awareness Scale (LEAS) is another self-report measurement and has been demonstrated no overlap with measures of negative effect (Lane and Schwartz, 1987; Subic-Wrana et al., 2005). Of note that TAS-20 and LEAS are not correlated well (Subic-Wrana et al., 2005). In addition, some researchers questioned whether self-report measures is appropriate to measure alexithymia and they recommend the use of multiple methods of measurement (Kooiman et al., 2002; Bagby et al., 2006). There has been various instruments developed such as observer-rated measures including the modified Beth Israel Hospital Psychosomatic Questionnaire (BIQ), the Bermond-Vorst Alexithymia Questionnaire (BVAQ) (Morera et al., 2005), and the Toronto Structured Interview for Alexithymia (TSIA) (Caretti et al., 2011). Differences in the evaluation method of Alexithymia are fundamentally problematic in interpreting the influence of alexithymia on clinical conditions. We need a consensus on suitable assessment of alexithymia in accordance with various study designs, including epidemiologic, exploratory, and clinical researches.

## Influence of alexithymia on FGIDs

Alexithymia may contribute to an increased severity of FGID or a poor outcome independent of anxiety and depression from the epidemiological studies listed in Table 1. What is the possible mechanism and clinical implication of this association between alexithymia and FGID?

Enhanced perception of visceral stimuli called visceral hypersensitivity is one of the key features of IBS (Drossman and Hasler, 2016). One hypothesis is that alexithymia may enhance the visceral hypersensitivity in IBS. High alexithymia patients often have a tendency to amplify somatic sensations (Porcelli and Todarello, 2007) and sustain the physiological component of emotion response systems (Lumley et al., 2007). The data that support this theory, though, are inconsistent. A somatosensory amplification score (SSAS) was positively correlated with an alexithymia score in patients with somatoform disorder (Tominaga et al., 2014) or those with psychosomatic illness (Nakao et al., 2002), but not in patients with FD (Jones et al., 2004). Healthy subjects with alexithymia showed less sensitivity to a heartbeat detection test (Murphy et al., 2017) and pain from heat exposure (Pollatos et al., 2015), but were hyper sensitive to visceral pain induced by rectal distention (Kano et al., 2003). The insula, which corresponds to the visceral sensory cortex, in patients with alexithymia was strongly activated by visceral pain (Kano et al., 2003, 2015) or from watching pictures of others experiencing pain (Moriguchi et al., 2007). In contrast, the insula was activated less by imagining others' pain (Bird et al., 2010). In the chronic pain conditions, in which a high prevalence of alexithymia has been reported, the association between alexithymia and pain intensity is not always clear (Di Tella and Castelli, 2016). It has been suggested that not only sensory component of pain but also affective component of pain may contribute to the relationship between alexithymia and chronic pain conditions (Di Tella and Castelli, 2016). It is an important issue to be clarified that alexithymia is related to the visceral hypersensitivity. Amplifying visceral or somatic sensation has several aspects: subjective evaluation of physiological sensation such as level of pain or accuracy of heartbeat, subjective believe of their physical condition as measured by questionnaires on the sensory system, and cognitive process such as a mismatch between the actual image of somatic/visceral sensation represented in the brain and the subjective predicted state. The mismatch between actual physiological state and prediction has been hypothesized as one of the pathophysiology of IBS (Mayer, 2011). In addition, the influence of alexithymia on visceral sensation is different between healthy subjects and pathological conditions. It is required to investigate how alexithymia contribute to these aspects over healthy and pathological conditions in a large sample population.

Another possible mechanism may be the influence of alexithymia on physiological stress system including autonomic nervous system (ANS) and hypothalamic-pituitary-adrenal (HPA) axis. These systems are main mediators of brain-gut interaction and alteration of these systems has been reported in FGIDs (Chang, 2011; Drossman, 2016; Kano et al., 2017). Subjects with high alexithymia showed lower skin conductance reactivity at baseline (Gaigg et al., 2016) and during emotional imaginary (Constantinou et al., 2014; Peasley-Miklus et al., 2016) and electrical stimulation (Starita et al., 2016), that indicates physiological hypo-arousal and ANS dysfunction in alexithymia. Cortisol response was increased during anticipation of stress associated with alexithymia (de Timary et al., 2008; Hua et al., 2014). Healthy individual with higher TAS-20 subscale, difficulty of identifying feelings score demonstrated increased adrenocorticotropic hormone response to corolectal distention (Kano et al., 2007). There may be direct association between alexithymia and these stress response system or possibly alteration of visceral sensation is a prerequisite of the change of stress response system.

In conclusion, alexithymia may contribute to an increased severity of FGID or a poor outcome measured by TAS-20. The empirical data may indicate that the association between FGIDs and alexithymia may not be explained simply by “somatosensory amplification,” but biased interpretation of their symptoms not based on appropriate bodily sensation. The physiological component of the emotional or stress response system may be altered; however, the direction of causation between these alterations and the alexithymic cognitive and affective style is not clear. The studies on the association between alexithymia and physiological aspect of FGID has been sparse. Future studies are required to make a consensus of measurement of alexithymia, and elucidate the physiological mechanism of link between alexithymia and FGID.

## Author contributions

MK: Drafting of the manuscript and critical revision of the manuscript; YE: Critical revision of the manuscript; SF: Critical revision of the manuscript.

### Conflict of interest statement

The authors declare that the research was conducted in the absence of any commercial or financial relationships that could be construed as a potential conflict of interest.
